# RNA-protein complexes and force field polarizability

**DOI:** 10.3389/fchem.2023.1217506

**Published:** 2023-06-22

**Authors:** Hanna Baltrukevich, Piia Bartos

**Affiliations:** ^1^ Faculty of Pharmacy, Jagiellonian University in Krakow, Kraków, Poland; ^2^ School of Pharmacy, Faculty of Health Sciences, University of Eastern Finland, Kuopio, Finland

**Keywords:** RNA, protein, simulation, molecular dynamics, polarization, force field

## Abstract

Molecular dynamic (MD) simulations offer a way to study biomolecular interactions and their dynamics at the atomistic level. There are only a few studies of RNA-protein complexes in MD simulations, and here we wanted to study how force fields differ when simulating RNA-protein complexes: 1) argonaute 2 with bound guide RNA and a target RNA, 2) CasPhi-2 bound to CRISPR RNA and 3) Retinoic acid-inducible gene I C268F variant in complex with double-stranded RNA. We tested three non-polarizable force fields: Amber protein force fields ff14SB and ff19SB with RNA force field OL3, and the all-atom OPLS4 force field. Due to the highly charged and polar nature of RNA, we also tested the polarizable AMOEBA force field and the ff19SB and OL3 force fields with a polarizable water model O3P. Our results show that the non-polarizable force fields lead to compact and stable complexes. The polarizability in the force field or in the water model allows significantly more movement from the complex, but in some cases, this results in the disintegration of the complex structure, especially if the protein contains longer loop regions. Thus, one should be cautious when running long-scale simulations with polarizability. As a conclusion, all the tested force fields can be used to simulate RNA-protein complexes and the choice of the optimal force field depends on the studied system and research question.

## 1 Introduction

Molecular dynamics (MD) simulations are routinely used to study the structure and dynamics of biomolecules at the atomistic level. Even though the models are by their very nature wrong in many ways, they are useful in showing us atomistic details of phenomena which cannot be directly observed experimentally (Berro, 2018). MD simulations have led to advances in drug and enzyme design and material science, and they have greatly increased our understanding of the interactions of biomolecules at the atomistic level.

During the last few years, RNA-protein complex simulations have started to appear in the literature (for example, Estarellas et al., 2015; Jiang et al., 2015; Kalia et al., 2015; Krepl et al., 2015; Krepl et al., 2021; Bhandare and Ramaswamy, 2016; Chang et al., 2016; Harikrishna and Pradeepkumar, 2017; Kong et al., 2017; Palermo et al., 2017; Bochicchio et al., 2018; Casalino et al., 2018; Kandeel and Kitade, 2018; Liu et al., 2018; Tan et al., 2018; Bissaro et al., 2020; Habibian et al., 2020; Saltalamacchia et al., 2020; Jing and Ren, 2022; Rinaldi et al., 2022). While this field is starting to gain interest, it is unfortunate to see that there are only a few studies published which use multiple force fields in studying RNA-protein complexes (Gallardo et al., 2022). The selection of force field and other simulation parameters depends on the studied system (Šponer et al., 2018), and thus some time should be spent testing suitable options for each study case. One could assume that polarizability of a force field could help in simulations containing RNA, as strong electrostatic interactions are in dominant role in these systems and one of the problems why it has been difficult to develop parameters for nucleic acids in the point-charge force fields (Zhang et al., 2018; Šponer et al., 2018; Cesari et al., 2019; Tucker et al., 2022). However, polarizable force fields are computationally demanding and thus a few orders or magnitude slower than the traditional force fields. In this study, we wanted to find if there are differences between traditional non-polarizable point-charge force fields and a polarizable force field when describing the RNA-protein complex interactions. A new polarizable water model for non-polarizable force fields (Xiong et al., 2022) was published during the preparation of this manuscript, and it was also included to illustrate a compromise between the extremes.

**FIGURE 1:**
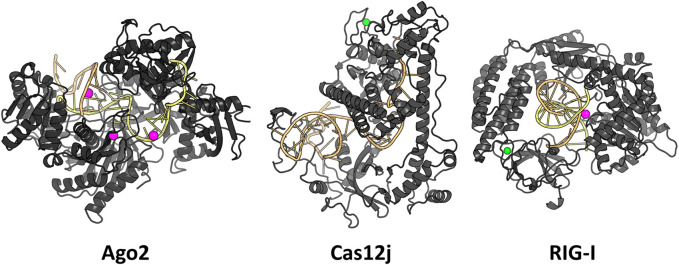
The studied RNA-protein complexes. Mg^2+^ ions are shown in magenta and Zn^2+^ ions in green.

We examined three RNA-protein complexes: 1) argonaute 2 with bound guide RNA and a target RNA (Schirle et al., 2014), 2) CasPhi-2 bound to CRISPR RNA (Pausch et al., 2021) and 3) Retinoic acid-inducible gene I C268F in complex with double-stranded RNA (Lässig et al., 2018) (Figure 1). For simplicity, we will refer to these complexes with the abbreviations Ago2, Cas12j, and RIG-I, respectively. Ago2 forms the base of the RNA-induced silencing complex (RISC) which inhibits gene expression by binding to the mRNA guided by the short siRNA (Wang et al., 2009). Cas12j is an RNA-guided nuclease that initiates CRISPR RNA complementary double-stranded DNA unwinding and cleavage in bacteriophages (Pausch et al., 2021). RIG-I is a cytosolic receptor that recognizes viral double-stranded RNA molecules as an immune sensor (Lässig et al., 2018). These complexes were chosen because they included only protein and RNA chains, were cytosolic, relatively small (contained less than 8,000 of modeled non-hydrogen atoms), had not too many missing residues in their structures and all of them had a large interaction surface between the RNA(s) and the protein.

We tested the MD simulations of the three RNA-protein complexes using three traditional non-polarizable force fields: Amber RNA force field OL3 (Zgarbová et al., 2011) with protein force fields ff14SB (Maier et al., 2015) and ff19SB (Tian et al., 2020), the all-atom force field OPLS4 (Lu et al., 2021, 4), the polarizable AMOEBA force field (Harger et al., 2017; Zhang et al., 2018) and the O3P polarizable water model (Xiong et al., 2022) with the ff19SB and OL3 force fields. The total simulation times for all systems were at least 1 µs. It is possible to run reasonable RNA-protein complex simulations with any of the tested force fields and none of them outperforms the others in all cases. However, especially when running hundreds of ns-scale simulations with the polarizable force fields or water model, one should pay attention to the structural integrity of the complex. Overall, we recommend testing of different force field combinations to find a setup that works for both your system of interest and research question.

## 2 Methods

### 2.1 System preparation

The complexes 4W5O (Ago2), 7M5O (Cas12j), and 6GPG (RIG-I) were downloaded from the PDB (Protein Data Bank) (Schirle et al., 2014; Lässig et al., 2018; Pausch et al., 2021). The missing nucleotides in the PDB model 4W5O were built manually in PyMOL (Version 2.5.1) (The PyMOL Molecular Graphics System, 2000). The missing loops in the protein structure were modelled in BioLuminate (Schrödinger, 2021.3), and afterwards the built loops were refined using Prime (Schrödinger, 2021.3) (Schrödinger Release 2020-4: Glide, Schrödinger, LLC, New York, NY, 2020). Both terminal phosphates were deleted from the ends of RNA molecules. The bounds ions (Zn^2+^ and/or Mg^2+^) were retained. The models were prepared in Protein Preparation Wizard (Schrödinger version 2021-3): the water molecules were deleted beyond 5.0 Å from het groups, the states of het groups were generated using Epik in pH 7.4 ± 2.0; the optimization of H-bonds was performed with PROPKA (Olsson et al., 2011; Søndergaard et al., 2011) in pH 7.4 followed by restrained minimization of them in OPLS4 (Lu et al., 2021, 4). The N-terminal of the proteins were not present in the crystal structures and they were omitted which is why Ago2 residue numbering starts from 22, Cas12j starts from 53 and RIG-I starts from 29. All systems were then converted to Amber atom names using the pdb4amber script in the AmberTools21.

### 2.2 Amber simulations–OL3, ff14SB and ff19SB

The Amber simulation systems were prepared with the tleap tool of AmberTools21 (Case et al., 2020). The RNA-protein complex was solvated with a water box with 10Å buffer using 0.15M NaCl solution with SPCE or OPC water model for ff14SB and ff19SB protein force fields, respectively. The ions used parameters specified with the corresponding water model, and the RNA was parameterized with the RNA force field OL3 (Zgarbová et al., 2011). To use the larger timestep (4 fs), the hydrogen masses were repartitioned to the connected atoms using ParmEd software (Shirts et al., 2017, 5).

For the ff19SB (Tian et al., 2020, 19) simulations, the minimization and equilibration steps were following: 1) all non-water atoms constrained, 2) heavy atoms constrained, 3) protein back bone constrained and 4) no constraints. The constraint force was 50 kcal/mol in the minimizations and 10 kcal/mol in the equilibration simulations. The minimizations 1–4 used the steepest descent algorithm with a maximum of 10,000 steps. The equilibration steps 1–3 consisted of 400 ps simulations, and step 4 was a 4,000 ps simulation. In the first equilibration step, the system was heated to 310 K. The temperature and pressure were maintained with Langevin thermostat and Berendsen barostat in the equilibration simulations. For the ff14SB, steps 2-4 were used in the minimization procedure and steps 2 and 4 for the equilibration runs of 20 ps and 2,000 ps, respectively.

During the production runs for each system the NPT ensemble was used: the 1.0 bar pressure was maintained with the help of Monte Carlo barostat and 310 K temperature was controlled by Langevin thermostat. Frames were recorded every 0.1 ns. The files were made ready for analysis by aligning and centering the complex, stripping away water molecules and writing the output in the format of xtc (compressed Gromacs trajectory) using cpptraj tool. The Amber production simulations were 4*500 ns in length for both force fields, resulting in 2 μs of total simulation time for each Ago2 complex. For the RIG-I and Cas12j systems, the ff14SB + OL3 simulations had a runtime of 4*300 ns, totaling 1.2 µs, and the ff19SB + OL3 simulations were of similar length than those of Ago2.

### 2.3 Amber simulations—ff19SB and O3P

The simulations with O3P water were prepared similarly as the ff19SB + OL3 simulations with OPC water model, with a few exceptions. The water box of O3P water is not equilibrated, and thus the box buffer was increased to 12Å. The increased buffer size lead to about the same number of water molecules that was present with the OPC simulations. The solvent box dimensions were then added using tleap to match the initial dimensions of the equilibrated OPC box from the previous simulations. To equilibrate this system more gently, the first equilibration simulation length was increased from 400 ps to 1 ns. The production run parameters were similar to the other Amber simulations.

### 2.4 Desmond simulations–OPLS4

The Ago2 complex was solvated with truncated octahedron water box with 15Å buffer using 0.15M NaCl solution with SPCE water model. The simulations in Desmond (Bowers et al., 2006) consisted of the default minimization and relaxation protocol and the production run applying Desmond default parameters as well: 2 fs timestep, ensemble class NPT with temperature 300 K controlled by Nose-Hoover chain thermostat and pressure 1.01325 bar maintained by Martyna-Tobias-Klein barostat, recording interval was set to 100 ps. The Desmond simulations of Ago2 complex were 8*250 ns in length, resulting in 2 μs of total simulation time. The smaller Cas12j and RIG-I complexes were placed in the cubic box with 12 Å buffer and solvated using 0.15M NaCl solution and SPCE water model. Both complexes were initially prepared with Protein Preparation Wizard and then processed with pdb4amber to preserve the naming of the atoms, which was important for the further cpptraj analysis. The Cas12j and RIG-I structures obtained in the following way were solvated and used for the Desmond simulations. However, due to absence of additional Protein Preparation Wizard step after using pdb4amber script, zero-order bonds to metals (Zn^2+^ and Mg^2+^) were omitted, and therefore the movements of these ions were unconstrained during simulation of Cas12j and RIG-I complexes in OPLS4 force field. After default minimization and relaxation protocol, the production run was parametrized using default settings with the temperature and pressure changed to comply with Amber simulations: NPT ensemble class, temperature 310 K, pressure 1.0 bar and recording interval each 100 ps. The Desmond simulations of the Cas12j and RIG-I complexes were run in 4*500 ns replicas, total 2 μs of simulation time.

### 2.5 OpenMM simulations–AMOEBA

The RNA-protein complex solvated with the Amber protocol was used to start the AMOEBA simulations in OpenMM (Eastman et al., 2017). To avoid clashes and high energy conformations in this slower force field, we used the last frame of an Amber trajectory as the starting conformation for the Ago2 simulations. The Cas12j and RIG-I simulations were run from the same initial conformation as the other simulations, as we determined no major equilibration issues as with the Ago2 system. The systems were first minimized using Verlet Integrator with 1 ps timestep for a maximum of 100 iterations. The production simulations were run with the default parameters (polarization method “mutual” and convergence threshold ε = 0.01) using Langevin integrator, 1/ps collision frequency and 2 fs timestep. We want to note that the rather large default convergence threshold has been changed to 0.00001 in later versions of OpenMM (our simulations were run in early 2022). We made a preliminary set of 10*10 ns simulations of Ago2 system to check for simulation stability, and then conducted a set of 10*100 ns simulations for all the systems that were used for the analysis. The simulation frames were saved every 0.02 ns.

### 2.6 Analysis of simulations–cpptraj

The resulting simulations were stripped from water atoms, wrapped into a single periodic box, centered around the protein and converted to.xtc-format to save disk space. These simulations are available in the Zenodo database. The stripped simulations were then analyzed using the in-house cpptraj scripts to calculate the RMSD, RMSF, PCA and hydrogen bond count. The simulation trajectories in .xtc format and their corresponding .pdb files have been uploaded into the Zenodo database under the following DOIs: 10.5281/zenodo.6605469 (Ago2 except for AMOEBA), 10.5281/zenodo.7694834 (Ago2 in AMOEBA), 10.5281/zenodo.7694878 (all Cas12j simulations) and 10.5281/zenodo.7695265 (all RIG-I simulations). The trajectories are wrapped into a single periodic boundary box, centered around the protein Cα atoms and the water molecules have been stripped out to conserve disk space.

## 3 Results

### 3.1 System flexibility and fluctuations–RMSD and RMSF

**FIGURE 2:**
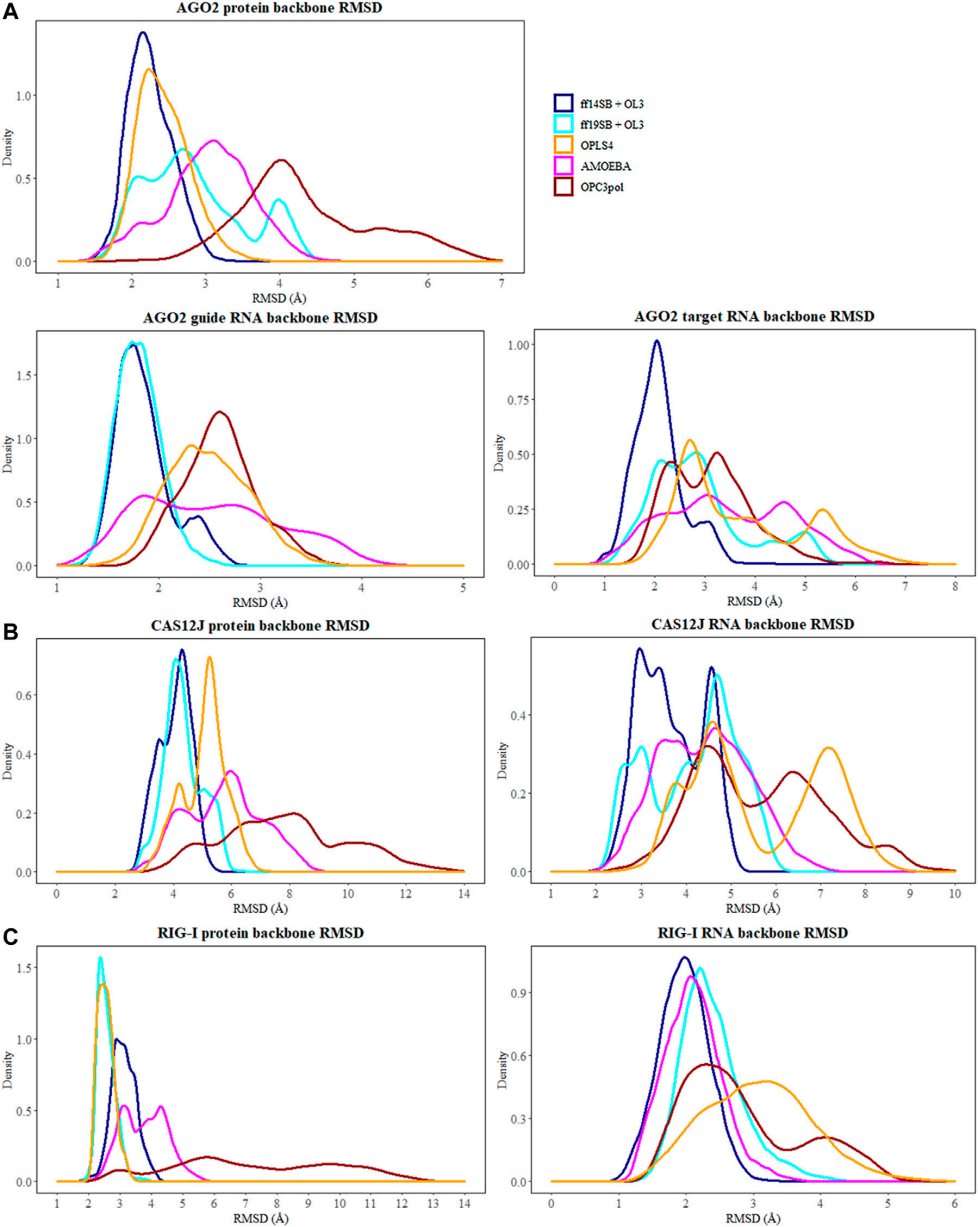
The distribution of RMSD values (Å) depicted at the kernel density estimate plots for: **(A)** Ago2 simulations; **(B)** Cas12j simulations; **(C)** RIG-I simulations. On each plot calculated RMSD values for the simulations performed in ff14SB + OL3 force field are represented as the dark blue line, ff19SB + OL3—cyan, OPLS4—orange, AMOEBA—magenta, O3P—dark red.

**FIGURE 3:**
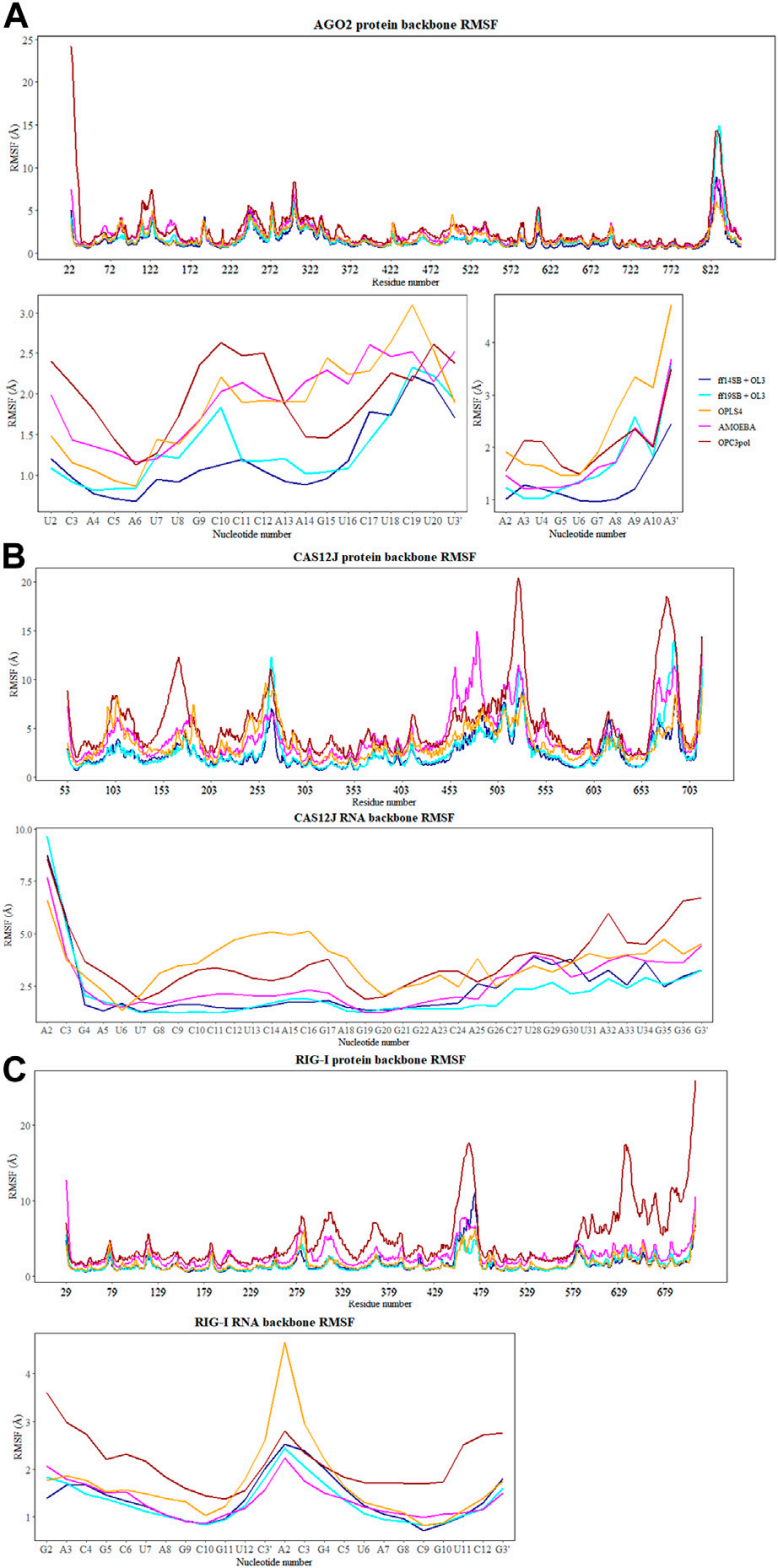
The RMSF values (Å) of Cα atoms of the protein and the P atoms of the RNA calculated for each aminoacid and nucleotide residue for: **(A)** Ago2 simulations; **(B)** Cas12j simulations; **(C)** RIG-I simulations. On each plot calculated RMSF values for the simulations performed in ff14SB + OL3 force field are represented as the dark blue line, ff19SB + OL3—cyan, OPLS4—orange, AMOEBA—magenta, O3P—dark red.

The flexibility of the protein and RNA strands was calculated using the root-mean-square deviation (RMSD) to the crystal structure. The results were calculated separately for the protein backbone (Cα, C, N, O atoms), guide RNA backbone (sugar + phosphate moieties) and target RNA backbone (sugar + phosphate moieties), and they are presented in Table 1. The distribution of RMSD values in simulations is depicted at the kernel density estimate plots, which are smoothed versions of the histogram in Figure 2, as well as at the time evolution graphs calculated from all the combined replicas in Supplementary Figure S2. The root-mean-square fluctuations (RMSF) of Cα atoms of the protein and the P atoms of the RNA are shown in Figure 3 and Supplementary Figures S3–S5.

**TABLE 1:** Average RMSD values (Å) in different simulations with corresponding standard deviations (SD).

Ago2	ff14SB + OL3	ff19SB + OL3	OPLS4	ff19SB + OL3+O3P_water_	AMOEBA
Protein backbone	2.2 ± 0.3	2.8 ± 0.7	2.4 ± 0.4 Å	4.4 ± 0.9	3.0 ± 0.6
Guide RNA backbone	1.9 ± 0.3	1.8 ± 0.2	2.5 ± 0.4 Å	2.6 ± 0.4	2.5 ± 0.7
Target RNA backbone	2.1 ± 0.5	2.9 ± 1.0	3.7 ± 1.3 Å	3.1 ± 0.9	3.5 ± 1.2

Cas12j	ff14SB + OL3	ff19SB + OL3	OPLS4	ff19SB + OL3+O3P_water_	AMOEBA
Protein backbone	4.0 ± 0.6	4.3 ± 0.7	5.1 ± 0.8	7.9 ± 2.2	5.7 ± 1.3
RNA backbone	3.7 ± 0.7	4.2 ± 1.0	5.7 ± 1.5	5.7 ± 1.4	4.4 ± 1.0

RIG-I	ff14SB + OL3	ff19SB + OL3	OPLS4	ff19SB + OL3+O3P_water_	AMOEBA
Protein backbone	3.1 ± 0.4	2.6 ± 0.3	2.6 ± 0.3	7.3 ± 2.6	3.8 ± 0.7
RNA A + B backbone	2.0 ± 0.4	2.4 ± 0.5	3.1 ± 0.8	2.9 ± 0.9	2.1 ± 0.4

**FIGURE 4:**
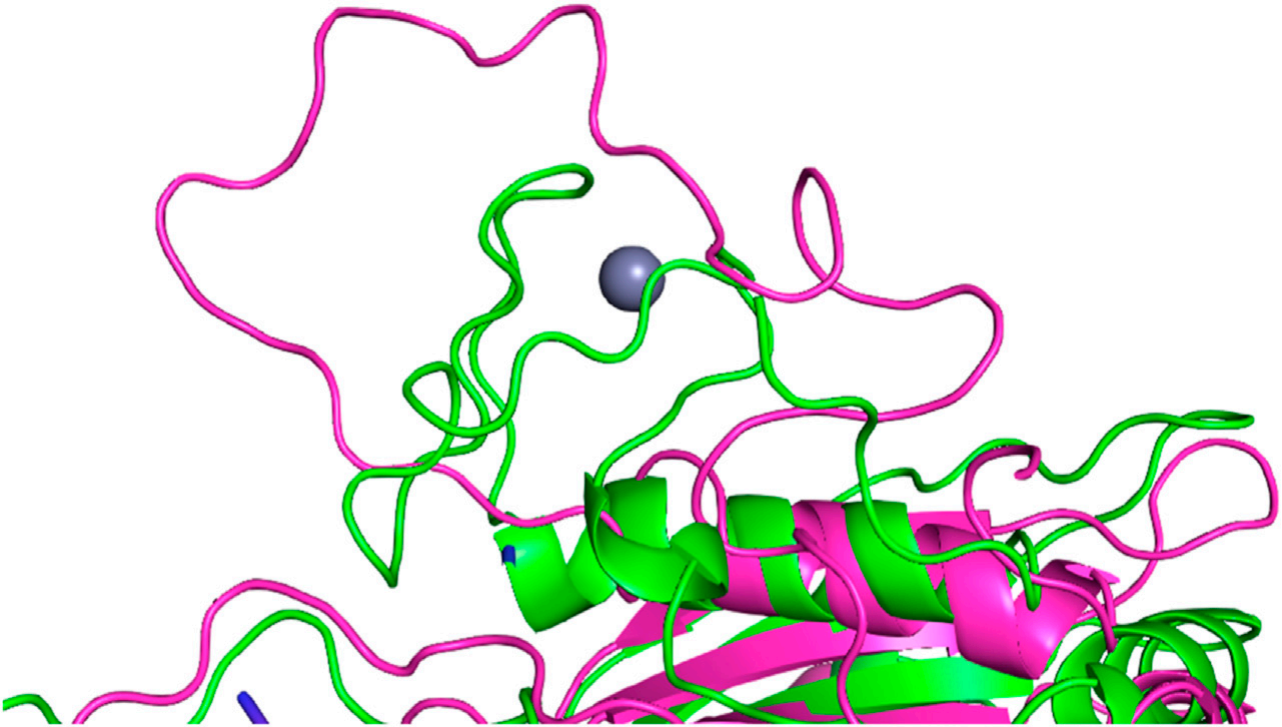
Structural integrity issue observed in the simulation with O3P water model simulations around the longer loop region (residues 660–700) in the Cas12j system. The starting structure is colored in green, the frame from the simulation in magenta. Zinc ion (in gray) bound by the mentioned above loop in the crystal structure unbinds in the first few ns of the simulation.

The simulations with the non-polarizable force fields display lower fluctuations than the simulations with the polarizable AMOEBA force field or the polarizable water model O3P. Indeed, the RMSD and RMSF values of O3P simulations clearly show that this water model leads to instability of the complex structure which can be confirmed by visual inspection of the trajectories. As the O3P simulations were run with similar parameters to ff19SB + OL3 simulation, it is likely that this instability is caused by the water model. However, in some of the AMOEBA simulations, similar structural integrity issues around the longer loop regions (residues 450–480 and 660–700) are observed on the Cas12j system (Figure 4).

### 3.2 Number of hydrogen bonds

**FIGURE 5:**
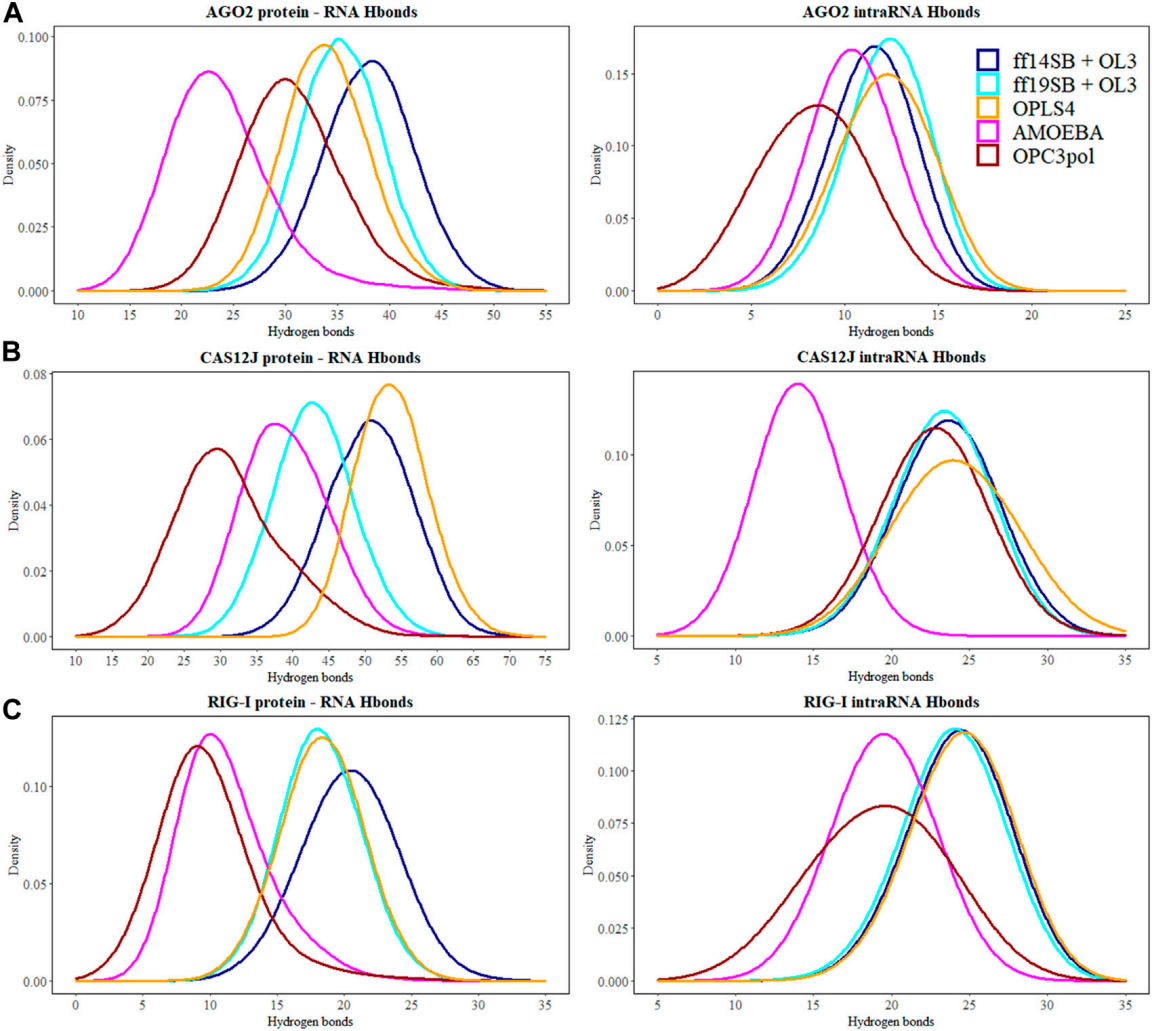
The number of hydrogen bonds between the protein and the RNA molecules are depicted at the kernel density estimate plots for: **(A)** Ago2 simulations; **(B)** Cas12j simulations; **(C)** RIG-I simulations. On each plot calculated H-bond values for the simulations performed in ff14SB + OL3 force field are represented as the dark blue line, ff19SB + OL3—cyan, OPLS4—orange, AMOEBA—magenta, O3P—dark red. Hydrogen bonds were calculated according to the definition of the strong bond assumed in this study—donor-acceptor distance <3.0 Å and angle D-H-A >135°.

The number of hydrogen bonds between the protein and the RNA and within RNA are shown in Table 2 and Figure 5 shows kernel density estimate plots. The hydrogen bond numbers for the crystal structures were calculated with both “strong” (donor-acceptor distance <3.0Å and angle >135°) and “weak” hydrogen bond definition (accordingly, <3.5Å and >120°), because the hydrogen bonding in the crystal structures was generally not agreeing with the stricter criteria. One possible reason for the low number of the hydrogen bonds in the initial structures might be relatively low resolution: 2.89 Å for RIG-I (X-ray) and 3.54 Å Cas12J (cryoEM) complexes. The discrepancy between the number of “strong” hydrogen bonds in the starting structure and the average from the simulations is especially clear in the case of Cas12J complex, which is the only cryoEM structure studied here. Ago2 complex is X-ray structure with 1.8 Å resolution, here the underestimated number of hydrogen bonds could be caused by the water mediating the hydrogen bonding, which could not be accessed by the calculation method we applied.

**TABLE 2:** The average number of hydrogen bonds within RNA and between RNA and protein in the simulation and their standard deviation (SD) during the simulations. Strong bond = Donor-acceptor distance <3.0 Å and angle >135°. *Weak bond = Donor-acceptor distance <3.5Å and angle >120°.

Intra-RNA	Crystal	ff14SB + OL3	ff19SB + OL3	OPLS4	ff19SB + OL3+O3P_water_	AMOEBA
Ago2	18 (20*)	11.5 ± 2.2	12.2 ± 2.1	12.2 ± 2.4	8.3 ± 2.8	10.4 ± 2.3
Cas12j	17 (22*)	23.6 ± 3.1	23.4 ± 3.0	24.2 ± 3.8	22.8 ± 3.3	14.1 ± 2.7
RIG-I	31 (34*)	24.3 ± 3.0	23.9 ± 3.1	24.5 ± 3.1	19.2 ± 4.2	19.5 ± 3.2

RNA-Protein	Crystal	ff14SB + OL3	ff19SB + OL3	OPLS4	ff19SB + OL3+O3P_water_	AMOEBA
Ago2	33 (38*)	38.0 ± 4.4	35.3 ± 4.0	33.9 ± 4.1	30.7 ± 5.0	23.7 ± 5.2
Cas12j	17 (40*)	50.7 ± 5.8	42.9 ± 5.6	53.7 ± 5.0	31.2 ± 7.5	39.0 ± 5.9
RIG-I	10 (19*)	20.4 ± 3.5	18.3 ± 3.0	18.4 ± 3.1	9.8 ± 3.7	11.2 ± 3.4

There are generally a few less intra-RNA hydrogen bonds observed in the Ago2 and RIG-I simulations than in the crystal structure (Table 2), and the difference is larger in the polarizable force field. In Cas12j simulations, the non-polarizable force fields display about same number of intra-RNA hydrogen bonds that was observed in the crystal structure, and there are less hydrogen bonds observed in the AMOEBA simulations.

The protein-RNA hydrogen bond numbers display more variation. In the Ago2 system, the non-polarizable force fields display about the same number of hydrogen bonds as the crystal structure, and the AMOEBA and O3P water simulations display less. In Cas12j system, ff14SB + OL3 and OPLS4 display more hydrogen bonds, ff19SB + OL3 and AMOEBA agree with crystal structure and O3P water simulations display less. In RIG-I simulations, the non-polarizable force fields agree with the crystal structure about the number of hydrogen bonds and the polarizable water model and AMOEBA display less hydrogen bonds.

These results indicate that the non-polarizable force fields stabilize the hydrogen bonding at the protein-RNA interface which has been reported before (Estarellas et al., 2015; Šponer et al., 2018; Gallardo et al., 2022).

### 3.3 Ions

Bound ions can affect the structure of the protein and the protein-RNA complex. In this study, Ago2 system had a bound Mg^2+^ ion, Cas12j had a bound Zn^2+^ ion and RIG-I system had bound Mg^2+^ and Zn^2+^ ions. Mg^2+^ ion is important for the catalytic activity of Ago2, however, in the complex we investigated, Mg^2+^ is inactivated by an inhibitory coordination to the main chain carbonyl of V598 (Schirle et al., 2014). Cas12j has a cofactor Zn^2+^ bound to the zinc finger, which together with nuclease domains is proposed to assist in the recruitment of DNA for the Cas12j cleavage (Cui et al., 2008; Lässig et al., 2018). In RIG-I Zn^2+^ coordination site is essential for signaling, while Mg^2+^ is involved in the ATPase activity (Huang et al., 2020; Pausch et al., 2021).

In the Ago2 simulations, the Mg^2+^ ion stays in place in all the tested force fields (Supplementary Figure S1). In the Cas12j simulations, the Zn^2+^ ion stays in place in ff14SB + OL3 and AMOEBA force fields, in the other ones it unbinds. In the RIG-I simulations, the Mg^2+^ ion stays in place in the ff14SB + OL3, ff19SB + OL3 and AMOEBA simulations and unbinds in the others. The Zn^2+^ ion is fluctuating more, but it displays similar behavior. Based on these results, one should use constraints with the structurally important ions to keep them in place. Due to an error in the preparation process of Cas12j and RIG-I systems for the OPLS4 force field, we did not include the default zero-order bonds to metals. However, this mistake made it possible for us to compare the stability of the ion binding without a bias in these systems.

### 3.4 PCA analysis

**FIGURE 6:**
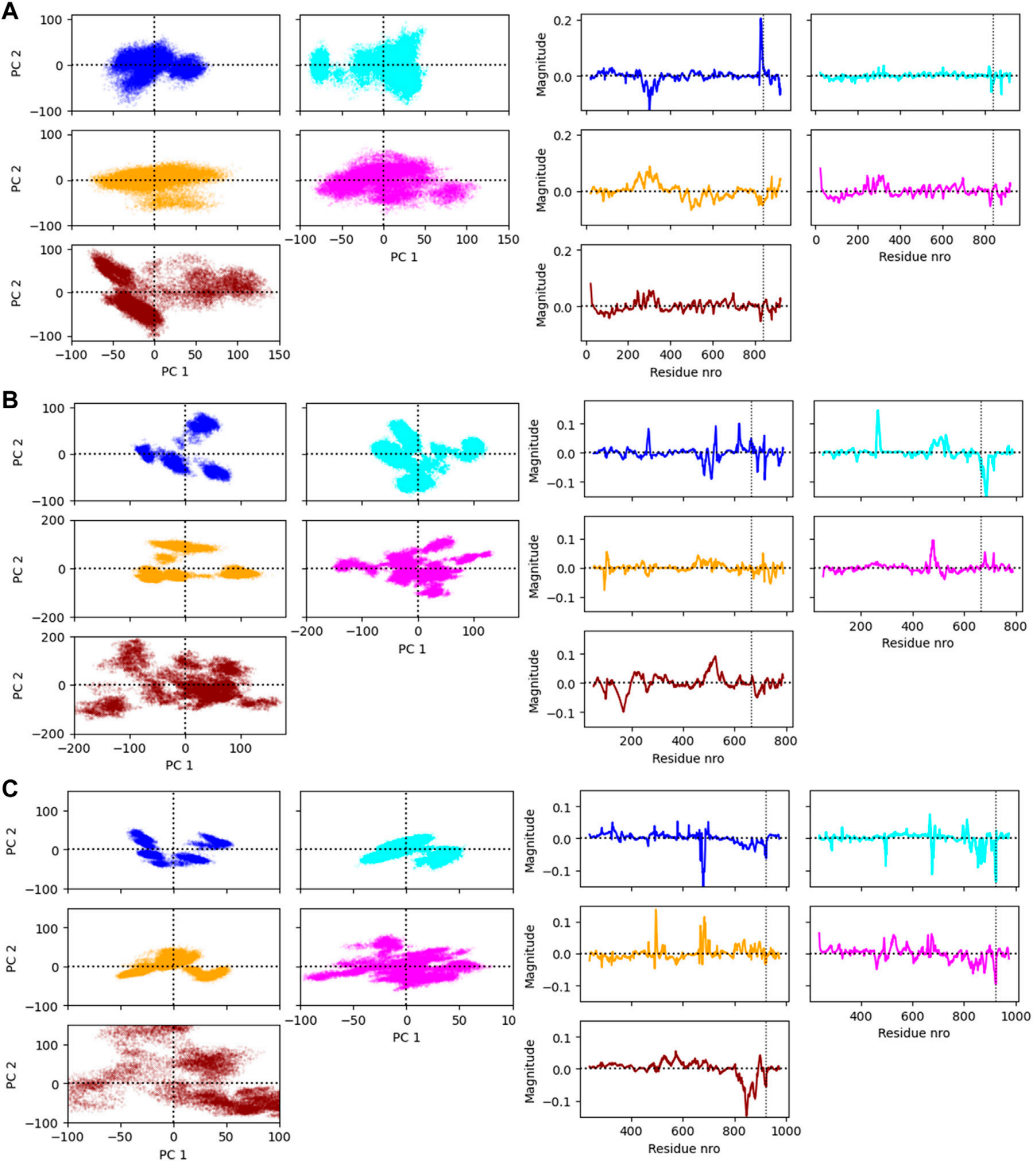
**(A)** The Ago2 simulation frames projected on the first two principal components (PC1 and PC2) and the loading values of the residues on PC1. **(B)** The Cas12j simulation frames projected on the PC1 and PC2 and the loading values of the residues on PC1. **(C)** The RIG-I simulation frames projected on the PC1 and PC2 and the loading values of the residues on PC1. The vertical line in the loading value plot indicates the start of the RNA. Note that there are two atoms analyzed per RNA nucleotide which changes the numbering of this region.

The principal component analysis was conducted for the coordinates of the CA atoms of the protein and the P and C4′ atoms of the RNA. The simulation frames plotted against the first two principal components (PC) are shown in Figure 6 on the left panel. On the right panel are shown the loadings of the atoms that were used to calculate the first PC. The PC loadings and weights show that the different force fields are generally not sampling a similar conformational space and the largest movements are not contributed by a certain part of the complex in all cases. The residue numbers that had the highest weights for the first PC are shown in Supplementary Table S1.

### 3.5 AGO2 results

AGO2 is a crucial component of the RNA-induced silencing complex (RISC) which when bound to an RNA molecule inhibits gene expression (Wang et al., 2009). The structure of AGO2 complex (4W5O) contains a protein of 859 residues (residues 1–21 not modeled), a guide RNA of 21 nucleotides and a target RNA of 11 nucleotides. The guide RNA binds to Ago2 mainly from the 5′ end which contains the seed sequence (nucleotides 2–7) which are crucial to the binding to the complementary mRNA strand (Kehl et al., 2017). The 3′ end of the guide RNA is bound to the most flexible part of Ago2, the PAZ domain (residues 235–348) (Jiang et al., 2015). The target RNA is mainly forming interactions with the guide RNA strand, with a few flanking nucleotides interacting with the Ago2 surface.

#### 3.5.1 Fluctuations of the AGO2 system

**FIGURE 7:**
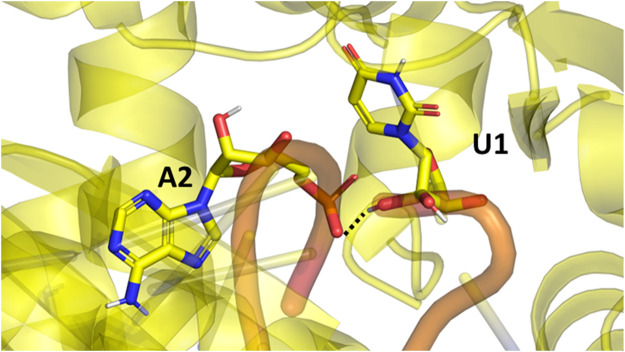
The extra hydrogen bond formed between the guide (U1) and target (A2) RNAs in the Ago2 system. This bond formed only in the OPLS4 simulations, and it was one of the reasons why OPLS4 simulations displayed lower RMSF and RMSD values.

In the Amber simulations, the protein is fluctuating more than either of the RNA strands. In the OPLS4 and AMOEBA force fields, the situation is the opposite, and the RNA fluctuates more than the protein. The fluctuations of the guide RNA are more pronounced in the polarizable force fields and around nucleotides 9–18 which are not tightly bound to the protein. Some of the RNA stabilization in OPLS4 force field is caused by an extra hydrogen bond forming between the guide RNA U1 and the target RNA A2 (Figure 7). For the protein, the longer loop regions around residues 110, 605 and 830 appear to fluctuate more in the Amber ff19SB + OL3 force field and the O3P water systems, but otherwise the trends in the RMSF graph are similar in all the tested force fields (Figure 2; Supplementary Figure S3). The longer loops around residues 110 and 830 are very much fluctuating in the O3P simulations but this does not lead to overall structural instability in these 500 ns simulations. The Mg^2+^ ion in the AGO2 system is tightly bound between the protein and the RNA and it remains in its position in all simulations in all force fields.

#### 3.5.2 Hydrogen bonds of the AGO2 system

The hydrogen bonds between the guide and target RNA base pairs remain stable in all tested force fields (Table 2; Figure 5). From these force fields, OPLS4 and ff19SB + OL3 match the crystal structure hydrogen bond count. In all the nonpolarizable force fields, there are on average more observed hydrogen bonds between the protein and the RNA than in the crystal structure. In the polarizable simulations, there are less hydrogen bonds than in the crystal structure. The complexes in polarizable conditions are not as compact as in the nonpolarizable simulations. The complexes open more in these simulations, which increases the distances of the hydrogen bonds beyond our cutoff distance and lowers the number of hydrogen bonds.

#### 3.5.3 Exploration of conformations in the AGO2 system—PCA

Figure 6 (right panel) shows the simulation frames are plotted against the first two PCs. In the case of AGO2, the distribution of PC1 and PC2 values are relatively similar within Amber force fields and OPLS4, and AMOEBA samples a slightly larger conformational space. O3P water simulations sample the largest conformational space, but these states are likely highly defined by the larger fluctuations of the terminals. Generally, there are two to three somewhat distinct conformational states observed in the PCA plots for the Ago2 system.

To observe which protein residues and RNA nucleotides have the highest effect on the first PC, we listed the top 10 residues based on their absolute PC weight (Figure 6A, right panel; Supplementary Table S1). The first PC is heavily affected by the flexible loop around residue 830 in the Amber and AMOEBA force fields. Except for ff19SB + OL3, high weights are observed on residues around 300 which contains the second most flexible region in the AGO2 system, the PAZ domain that binds the 3′ end of the RNA. ff19SB + OL3 and O3P simulations are the only simulations where one or two RNA atoms make it into the top weighing residues for PC1. In O3P simulations, the highest contributions are from either of the terminals of the protein which are also fluctuating more than in the other conditions. The N-terminal residues are also important for the AMOEBA PC1.

### 3.6 Cas12j results

Cas12j is an RNA-guided nuclease that bacteriophages use for DNA cutting and genome editing (Pausch et al., 2021). The structure of Cas12j (7M5O) contains a protein of 763 residues (residues 1–52 and 717–763 unmodeled) and a single-stranded crRNA molecule of 45 nucleotides. In addition, there is a Zn^2+^ ion bound in the zinc finger part of the protein. The crRNA forms a hairpin loop with the first 23 nucleotides, and it continues as a single stranded spacer region that is used to recognize the DNA sequence to be cut.

#### 3.6.1 Fluctuations of the Cas12j system

The Cas12j system has overall higher RMSD values than the Ago2 system. The protein is not as compact and globular as Ago2 and there is more freedom of movement also for the RNA. The higher RMSD values do not necessarily mean that the system is fluctuating more, as they could be a result of the initial structure being further away from a local energy minimum. Then, in the simulations the system adopts an energetically more favorable conformation which is by RMSD further away from the initial one and fluctuates around that conformation. As the deviations of the RMSD values are like those of Ago2, this seems to be the case with Cas12j. The Amber force fields display the least fluctuating protein and RNA, and the largest RMSD values are observed in the AMOEBA and O3P systems for the protein and in the OPLS4 and O3P systems for the RNA. The Zn^2+^ ion stays in place in ff14SB + OL3 and AMOEBA force fields, in the other ones it unbinds. This leads to overall structural instability which is most obvious in the O3P simulations (Figure 4).

#### 3.6.2 Hydrogen bonds of the Cas12j system

There are 22 intra-RNA hydrogen bonds in the Cas12j crystal structure (Table 2). The simulations have a similar number of hydrogen bonds, except for AMOEBA where the hydrogen bond number is lower. This result is a combination of slight structural instability issues and the generally more open and relaxed conformation of the complex that leads the hydrogen bonds to be longer than our relatively strict criteria.

There are 40 hydrogen bonds between the protein and the RNA in the preprocessed starting structure. From these force fields, the ff19SB + OL3 and AMOEBA closely match the initial hydrogen bond count. Like the Ago2 systems, in all the nonpolarizable force fields, there are on average more observed hydrogen bonds between the protein and the RNA than in the crystal structure. In the O3P water simulations where there are structural integrity issues, the number of hydrogen bonds drops by 9.

#### 3.6.3 Exploration of conformations in the Cas12j system—PCA

The plots of PC1 and PC2 values show that Cas12j system adopts clearer distinct conformational states than the Ago2 system (Figure 6B, left panel). There are four to five different states in all the tested force fields. The PC analysis of O3P simulations shows a larger sampling of the conformational space which is due to the structural instability.

Based on the loadings graphs, the different force fields are not sampling similar conformational space. The Amber ff14SB + OL3 and ff19SB + OL3 force fields have the highest weights around the residues 265 and 510, and the 5′ end of the RNA (Figure 6B, right panel; Supplementary Table S2). The loop of the RNA hairpin is located close to the flexible oligonucleotide binding domain (OBD) protein region around residue 265 and the movement of these interacting parts are connected. The first 3 nt of the 5′ end of the RNA are moving rather freely and not interacting with the protein which might explain their higher effect on the first PC in these force fields. Residue 510 is part of a long loop (508–537) which forms part of the second recognition domain (RecII) which can interact with the 3′ end of the RNA. This RecII domain is also visible on the loadings plots of the polarizable force fields. The highest loadings in the OPLS4 force field are observed around the middle region of the RNA and around residue 100 which forms a loop in the first recognition domain (RecI).

### 3.7 RIG-I results

RIG-I is a protein that binds cytosolic viral RNA and ATP to trigger an immune response (Lässig et al., 2018). In this study, we used the C268F variant of RIG-I that can trigger the immune response without ATP. The crystal structure of RIG-I consists of a protein of 922 residues (residues 1–239 and 823–925 not modeled) which surrounds two identical RNA chains of 13 nucleotides that are bound into a double helical structure. There is also one Zn^2+^ and 1 Mg^2+^ ion bound in the complex.

#### 3.7.1 Fluctuations of the RIG-I system

Similarly to the Ago2 systems, the protein is moving more than the RNA in the simulations (Table 1). The RMSD values are slightly higher than with the AGO2 system and lower than with the Cas12j system, indicating that the system remains relatively close to the initial conformation of the crystal structure. The RMSD values observed in the non-polarizable force fields are comparable to each other and AMOEBA simulations displays slightly elevated values. The O3P water model leads to structural integrity issues of the protein, starting from terminals and longer loop regions, similarly to the Cas12j system. This can be easily seen in Figure 2C where the distribution of protein RMSD values in the O3P simulations is very wide, whereas the RMSD in the other simulations remain comparable to the other protein systems. The RIG-I system has both a Zn^2+^ and a Mg^2+^ ion which behave differently (Supplementary Figure S1). The Zn^2+^ ion unbinds from the protein in all the simulations except some of the AMOEBA simulations. The Mg^2+^ ion is stable, and it stays in its place in all the simulations except for OPLS4.

#### 3.7.2 Hydrogen bonds of the RIG-I system

There are 31 strong RNA-RNA hydrogen bonds in the RIG-I crystal structure (Table 2). All simulations display at least six less hydrogen bonds on average, the polarizable O3P water model and AMOEBA both display on average less than 20 hydrogen bonds in total. This loss of hydrogen bonds is explained by the one or two base pairs breaking from each other at the end that is not bound on the protein.

There are 19 weak hydrogen bonds between the protein and the RNA in the preprocessed starting structure. The non-polarizable force fields closely match the hydrogen bond amount observed in the initial structure. The hydrogen bond number is significantly lower in the simulations with the polarizable water model O3P or the AMOEBA force field. This trend is similar to the other two studied systems.

#### 3.7.3 Exploration of conformations in the RIG-I system—PCA

The PCA shows two to four somewhat distinct conformational states for the RIG-I system in the non-polarizable force fields (Figure 6C, left panel). The states are not very distinct in AMOEBA or O3P water model simulations and the O3P simulations sample a large conformational space due to the structural integrity issues.

The weights of the first PC (Figure 6C, right panel) display that the nonpolarizable force fields relatively well agree which parts of the RIG-I complex contribute most to the different conformations. All non-polarizable force fields display larger magnitude around residue 685 which is part of the long loop (residues 676–720) residing close to the catalytic site (around residue 268 which is mutated in our variant C268F) which normally binds ATP and a Mg^2+^ ion. The movements of this loop are required to access the catalytic site, and based on these simulations it seems that this loop is fluctuating a lot possibly to accommodate the ATP and Mg^2+^ binding. The ff19SB + OL3 has higher weight close to residue 439 which is a part of a smaller loop on the other side of the catalytic site. In the OPLS4 force field, higher weights are observed around residue 500 which is a smaller loop region connecting two helices. In all the non-polarizable force fields, the C terminus of the protein gets high weights. The C terminus of RIG-I contains long loop regions without many helical or sheet structures which makes it more structurally unstable than the rest of the protein. Also, it binds the Zn^2+^ ion which unbinds in many of the simulations leading to more structural instability. This is why the C terminal residues also get high weights in the polarizable force fields which tend to sample more conformations in these less structured regions. The effect of the RNA atoms for the PCA analysis is negilible.

## 4 Discussion

All the studied force fields can be used with RNA-protein complexes.

All force fields produced reasonable simulations without major artefacts that could be traced back to the parameterization of the force fields. The RNA-protein complexes are chemically and biologically special systems, for which there are no specifically tailored parameters in any currently available force field. Proteins and nucleic acids are chemically different which has led to force fields being developed for them separately or making compromises in the form of all-atom force fields. The comparison of force fields presented here is not perfect as it is missing CHARMM36 force field that has been discussed recently in the context of RNA-protein complexes (Gallardo et al., 2022). This, and other weaknesses are discussed in Supplementary Material Part 6.

### 4.1 Force field selection depends on the studied system

As described previously (Krepl et al., 2015), the choice of the protein and RNA force field depends on many factors of the studied system. There was no single force field that would outperform others in all of our studied complexes, and thus the selection of the force field should happen based on the studied system and the research question. Especially the flexibility of the system affected the force field performance in this study: the flexible Cas12j system displayed some structural instability with the AMOEBA force field and O3P water model, whereas this was not as obvious with the more stable and globular Ago2 system. The instability was most notably observed in the O3P water model simulations, where even some of the Ago2 systems displayed large fluctuations and lower number of hydrogen bonds. It is possible that the very recently published fine-tuned van der Waals parameters of the AMOEBA force field (Jing and Ren, 2022) would alleviate instability issue in the AMOEBA force field.

### 4.2 Structurally important ions might need constraints in any force field

Many protein RNA-complexes contain ions, which are crucial for protein activity. Ions as charged particles pose a challenge for the non-polarizable force fields which could be observed as the ions unbinding. The issues of ions in nonpolarizable force fields have been described many times before (for example, Leontyev and Stuchebrukhov, 2011; Bedrov et al., 2019; Kurki et al., 2022), and there are no easy solutions. Seeing that the ions sometimes unbind also in the polarizable force fields, the only feasible solution is to constrain the structurally important ions positionally unless one especially wants to study the ion unbinding.

### 4.3 Polarizability is computationally demanding and might induce structural instability

Using the polarizable force field comes with a significant computational cost. While the ff19SB + OL3 simulations achieved a simulation speed of ∼300 nsday for the largest (Ago2) complex, the AMOEBA simulations only reached a simulation speed of ∼4 ns/day. All simulations were run in a similar environment, on a single NVIDIA Volta V100 GPU on CSC’s supercomputer Puhti. Due to this significant increase in computational cost, in some cases it might be more beneficial to use non-polarizable force fields with an enhanced sampling method to get reasonably accurate results. The polarizable water model O3P only minorly decreased the simulation speed (∼280 ns/day).

The recently published polarizable water model O3P (Xiong et al., 2022) was not a very good choice for RNA-protein complex simulations. Just changing the water model without changing the simulation parameters or force fields lead to disorganized protein structures that were most prominent on Cas12j system. This water model is not likely the best choice for RNA-protein complexes before some further optimization of its parameters.

### 4.4 The nonpolarizable force fields stabilize the complexes and might hide rare conformational states

Even though both Amber simulation sets used the same RNA force field, the complexes behaved slightly differently with the different complexes. It is known that the Amber ff14SB force field underestimates helicity which in connection to 3-point water models leads to overly compact protein structures (Tian et al., 2020). This behavior is enhanced when RNA is bound to the protein, as the electrostatic interactions with the RNA backbone make the complex even more compact. Changing the protein force field to ff19SB and the water model to the 4-point OPC alleviate the problem, as the results show more flexible protein backbone movement (Onufriev and Izadi, 2018). OPLS4 and ff14SB + OL3 simulation sets used the older 3-point water model SPC/E. Even in the case of OPC water model, the electrostatic interactions of RNA with the protein and the other RNA strand are strong which leads to less freedom of movement to all biomolecules. The strong charge-charge interactions are a known issue in non-polarizable force fields (Yoo and Aksimentiev, 2016; Aksimentiev, 2018; Duboué-Dijon et al., 2020; Gallardo et al., 2022), for which there are no easy solutions.

Generally, only adjusting Lennard-Jones parameters, vdW parameters or changing the water model are proposed to help with the issue of strong electrostatic interactions (Nerenberg et al., 2012; Chen and García, 2013; Tan et al., 2018; Duboué-Dijon et al., 2020; Tian et al., 2020, 19). Electronic continuum correction approach can be used to adjust the charges of ions to enhance the ion representation in non-polarizable force fields (Duboué-Dijon et al., 2020). This methodology could be partially also employed on proteins, where the charged side chains are relatively distant from the backbone. However, in the case of RNA, the ribose-phosphate backbone is heavily charged and any simple modifications to the point charges would compromise the description of the backbone dihedrals which are directly related to the point charges.

## 5 Conclusion

Even though extensive effort has been put to parameterize the force fields for RNA and proteins, the RNA-protein complex simulations remain problematic because they need to provide reasonable interactions at the interface of these two chemically different molecules. This study further confirms that the force field and other simulation parameters selection is always dependent on the studied system. Based on our results all the tested non-polarizable force fields can be used to simulate RNA-protein complexes. However, all the non-polarizable force fields tended to make the complexes very compact which might prevent the formation of some biologically relevant conformations. To avoid this, and when the computational cost is not an issue, the polarizable force field AMOEBA could be preferred, but the polarizable O3P water model cannot be recommended for RNA-protein complex simulations. We perceive that the polarizable force fields are the future of biomolecular simulations also beyond the RNA-protein complexes after sufficient development of software and hardware makes them a computationally reasonable alternative.

## Data Availability

The datasets presented in this study can be found in online repositories. The names of the repository/repositories and accession number(s) can be found in the article/Supplementary Material.
