# Supplementary material for: RNA-protein complexes and force field polarizability
Source: Front Chem. 2023 Jun 22;11:1217506. doi: 10.3389/fchem.2023.1217506 (PMC10323139; doi:10.3389/fchem.2023.1217506)
Supplement: Supplementary file 1 [file DataSheet1.docx]

RNA-protein Complexes and Force Field Polarizability

**Supporting Information**

Hanna Baltrukevich^1^, Piia Bartos^2^*

^1^Jagiellonian University in Krakow, Faculty of Pharmacy, ul. Gołębia 24, 31-007 Kraków, Poland

Current affiliation: Department of Pharmaceutical Chemistry, Philipps-University of Marburg, 35032 Marburg, Germany

^2^University of Eastern Finland, School of Pharmacy, Faculty of Health Sciences, Yliopistonranta 1C, 70210 Kuopio, Finland

*** Correspondence:**Corresponding Author
Piia.Bartos@uef.fi

Contents

[1 RMSD of the bound ions 2](#_Toc137452065)

[2 Time evolution of RMSD 3](#_Toc137452066)

[3 RMSF of the protein in AGO2 5](#_Toc137452067)

[4 RMSF of the protein in CAS12J 6](#_Toc137452068)

[5 RMSF of the protein in RIG-I 7](#_Toc137452069)

[6 Top 10 PCA weighing residues 8](#_Toc137452070)

[7 Study limitations 9](#_Toc137452071)

# 1 RMSD of the bound ions

**
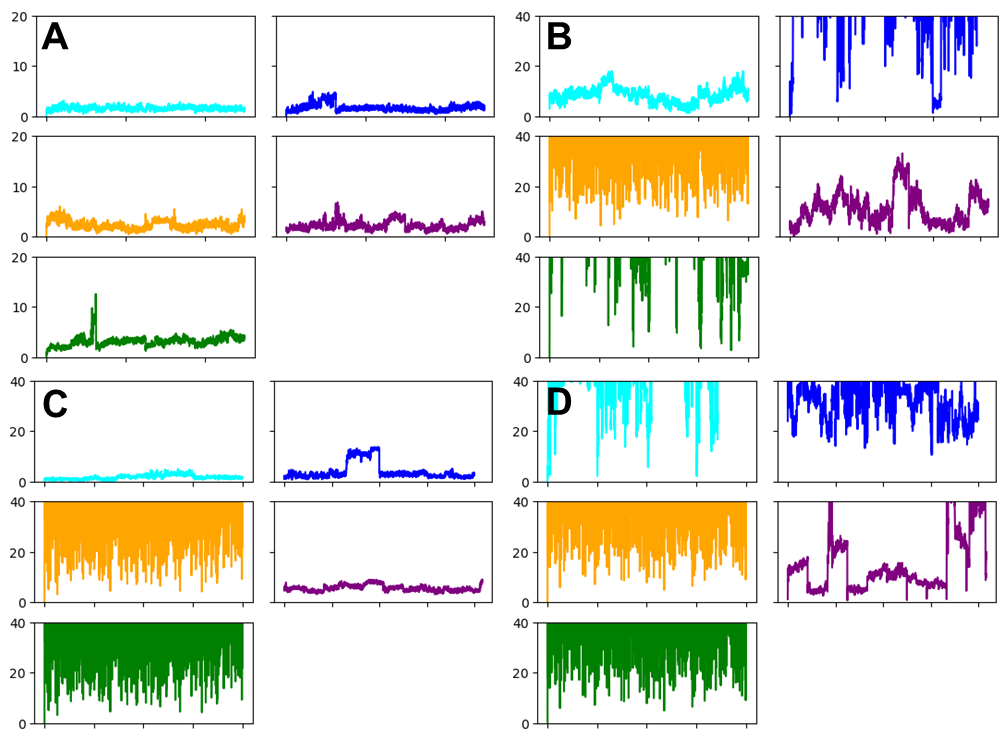
**

**Figure S1.** A) The RMSD of the bound Mg ion in the Ago2 system. B) The RMSD of the bound Zn ion in the Cas12j system. C) The RMSD of the bound Mg ion in the RIG-I system. D) The RMSD of the bound Zn ion in the RIG-I system.

# 2 Time evolution of RMSD

**
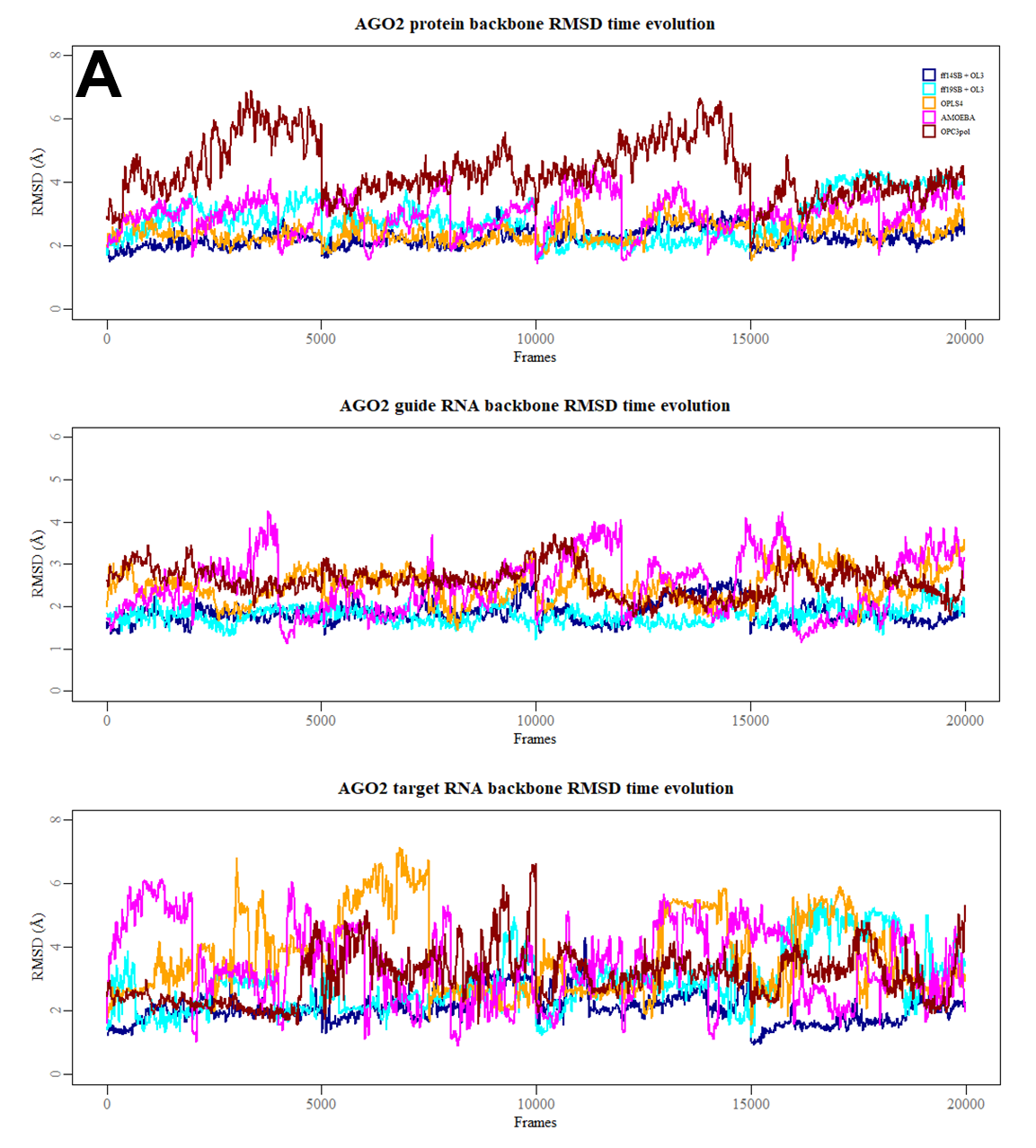
**


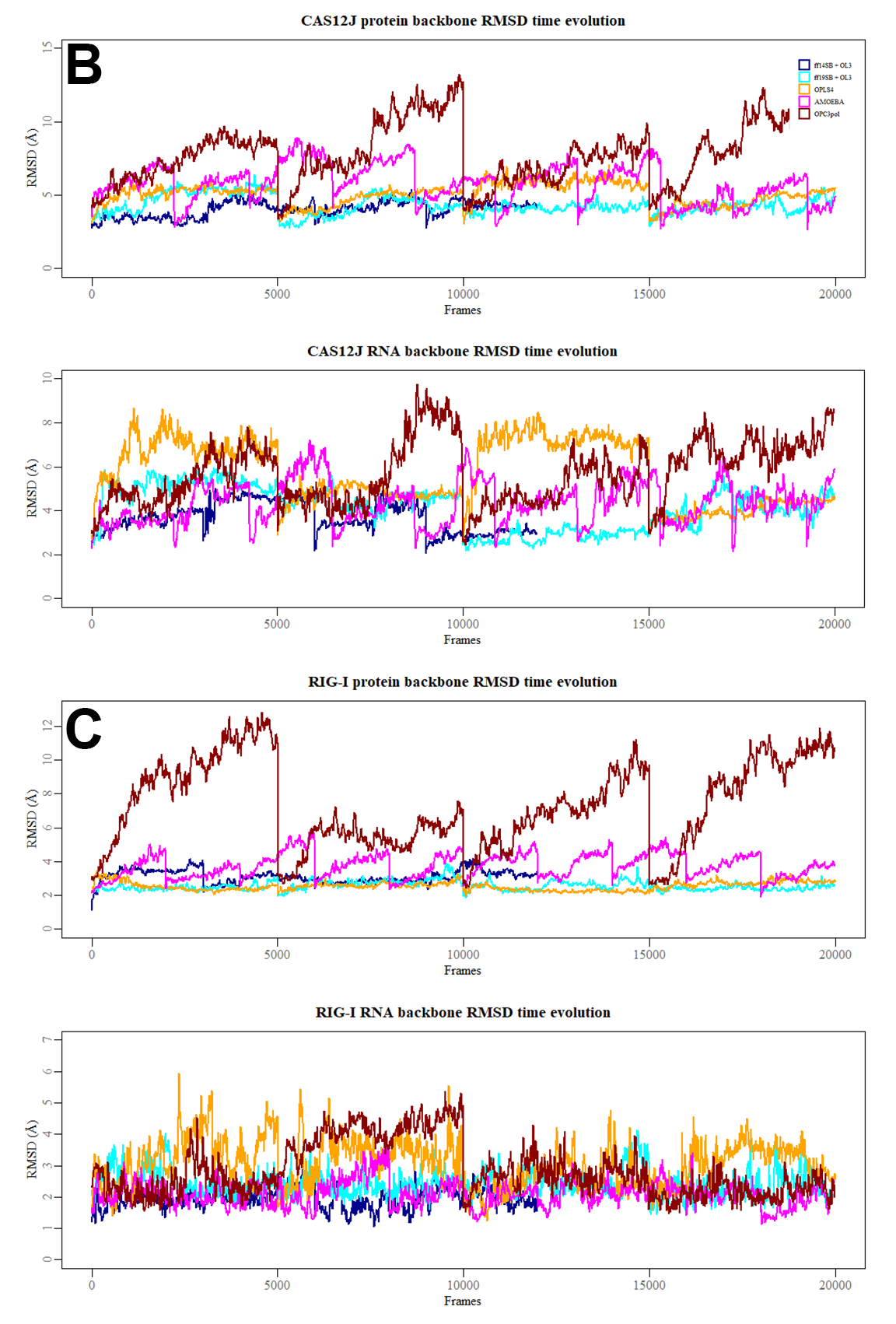


**Figure S2.** The RMSD values (Å) of Cα atoms of the protein and the P atoms of the RNA calculated for each aminoacid and nucleotide residue from all combined replicas for A) Ago2; B) Cas12j; C) RIG-I systems. The mean value was taken from each 10 frame for ff14SB, ff19SB, OPLS4 and OPLC3-pol, and each 25 frames for AMOEBA simulations. RMSD values for the ff14SB + OL3 force field are represented as the dark blue line, ff19SB + OL3 – cyan, OPLS4 – orange, AMOEBA – magenta, OPC3-pol – dark red.

# 3 RMSF of the protein in AGO2


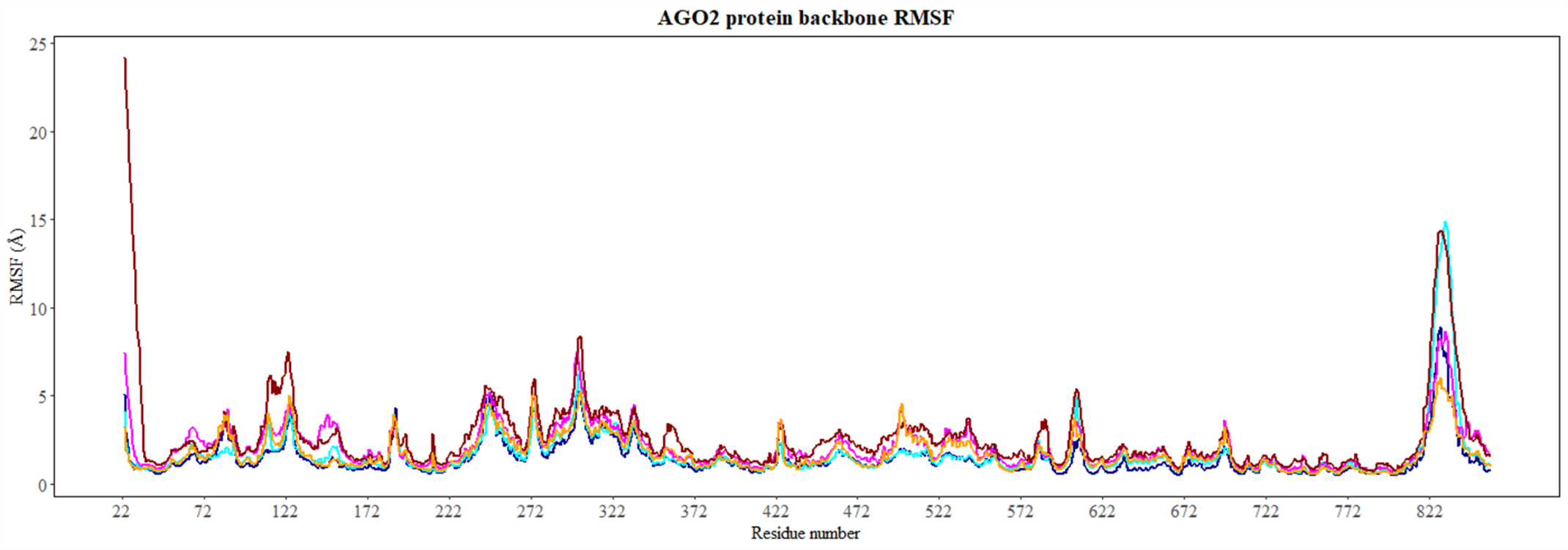
**Figure S3.** The RMSF values (Å) of Cα atoms of the protein and the P atoms of the RNA calculated for each aminoacid and nucleotide residue for Ago2 simulations. On each plot calculated RMSF values for the simulations performed in ff14SB + OL3 force field are represented as the dark blue line, ff19SB + OL3 – cyan, OPLS4 – orange, AMOEBA – magenta, OPC3-pol – dark red.

# 4 RMSF of the protein in CAS12J

**Figure S4.** The RMSF values (Å) of Cα atoms of the protein and the P atoms of the RNA calculated for each aminoacid and nucleotide residue for Cas12j simulations. On each plot calculated RMSF values for the simulations performed in ff14SB + OL3 force field are represented as the dark blue line, ff19SB + OL3 – cyan, OPLS4 – orange, AMOEBA – magenta, OPC3-pol – dark red.


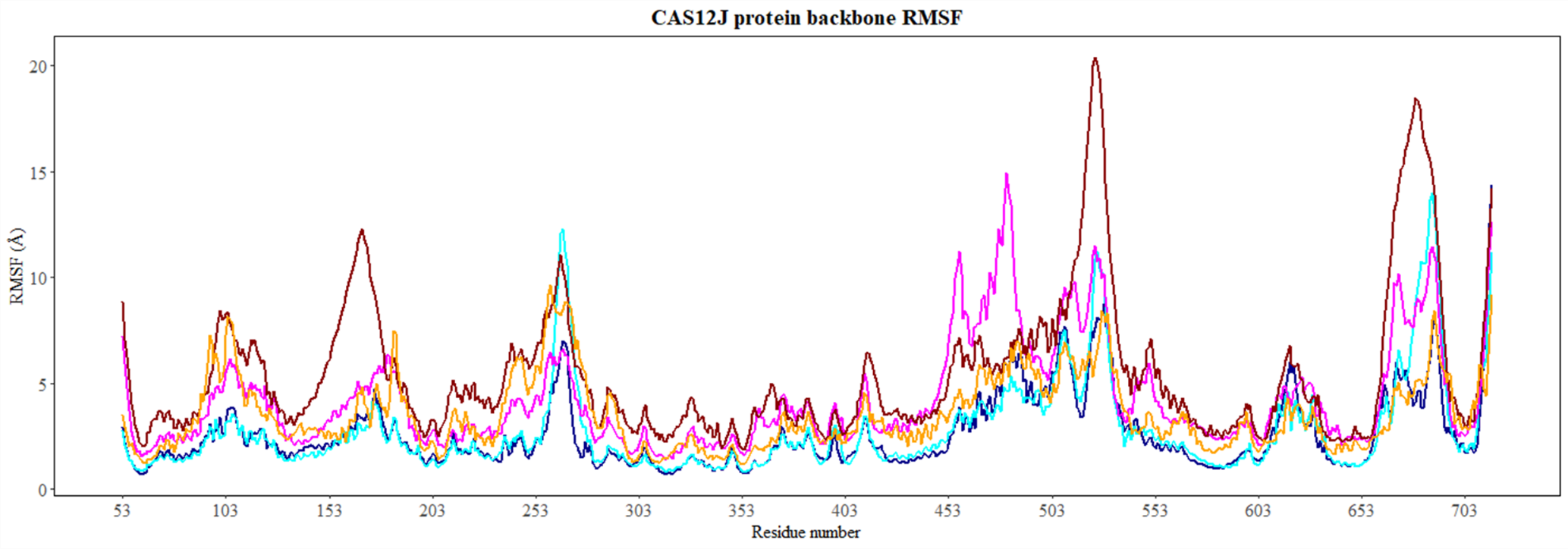


# 5 RMSF of the protein in RIG-I

**Figure S5.** The RMSF values (Å) of Cα atoms of the protein and the P atoms of the RNA calculated for each aminoacid and nucleotide residue for RIG-I simulations. On each plot calculated RMSF values for the simulations performed in ff14SB + OL3 force field are represented as the dark blue line, ff19SB + OL3 – cyan, OPLS4 – orange, AMOEBA – magenta, OPC3-pol – dark red.


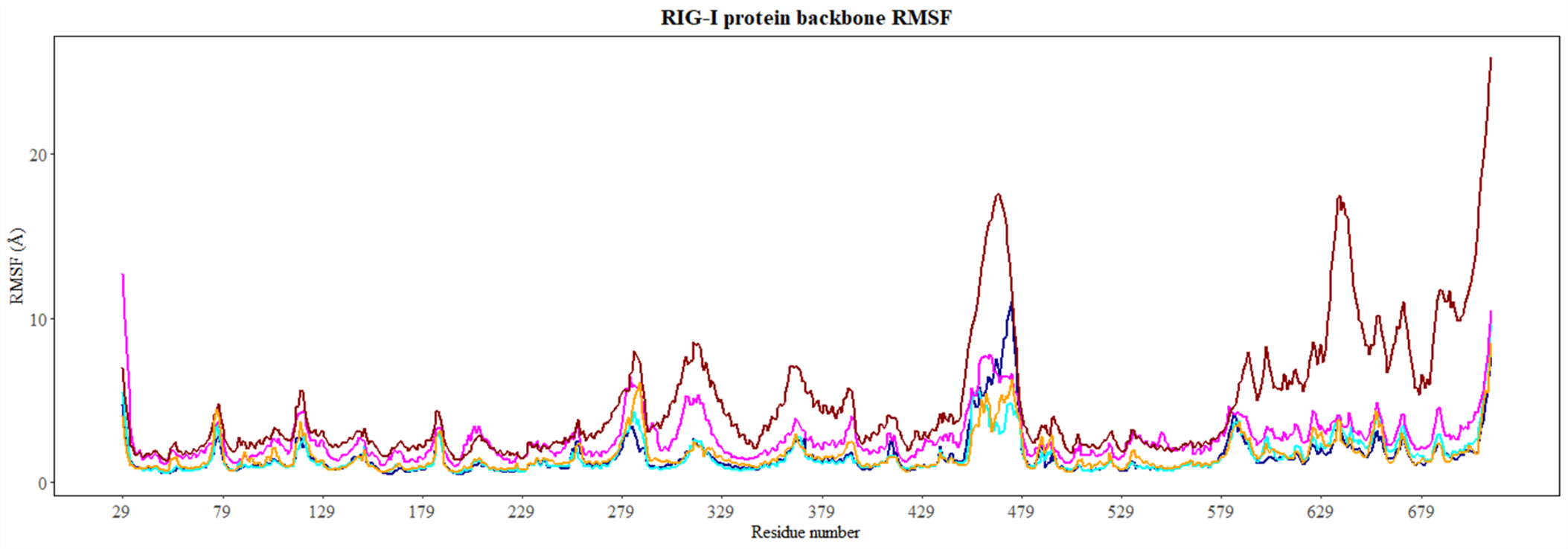


# 6 Top 10 PCA weighing residues

**Table S7.** The ten residue numbers that had the highest weights in the PC1.

|  |  | **Ago2** |  |  |
| --- | --- | --- | --- | --- |
| **fff14SB** | **ff19SB** | **OPLS4** | **AMOEBA** | **O3P** |
| 832 | 322 | 252 | 22 | 880 |
| 831 | 323 | 286 | 23 | 30 |
| 830 | 821 | 287 | 24 | 29 |
| 826 | 879 | 305 | 244 | 25 |
| 828 | 834 | 303 | 298 | 27 |
| 829 | 831 | 291 | 299 | 28 |
| 300 | 833 | 302 | 314 | 23 |
| 825 | 832 | 301 | 317 | 24 |
| 827 | 835 | 304 | 319 | 26 |
| 301 | 880 | 498 | 827 | 22 |
|  |  | **Cas12j** |  |  |
| **fff14SB** | **ff19SB** | **OPLS4** | **AMOEBA** | **O3P** |
| 265 | 264 | 94 | 473 | 168 |
| 508 | 265 | 95 | 475 | 167 |
| 510 | 266 | 102 | 476 | 169 |
| 526 | 681 | 103 | 477 | 166 |
| 527 | 682 | 104 | 478 | 170 |
| 616 | 683 | 709 | 479 | 165 |
| 617 | 684 | 715 | 480 | 164 |
| 618 | 685 | 737 | 481 | 523 |
| 619 | 686 | 738 | 482 | 525 |
| 716 | 687 | 739 | 483 | 524 |
|  |  | **RIG-I** |  |  |
| **fff14SB** | **ff19SB** | **OPLS4** | **AMOEBA** | **O3P** |
| 676 | 259 | 495 | 239 | 843 |
| 677 | 438 | 496 | 674 | 844 |
| 678 | 439 | 497 | 675 | 845 |
| 679 | 616 | 498 | 835 | 846 |
| 680 | 628 | 669 | 879 | 847 |
| 681 | 682 | 683 | 920 | 848 |
| 682 | 683 | 684 | 921 | 849 |
| 683 | 684 | 685 | 922 | 850 |
| 684 | 685 | 687 | 923 | 851 |
| 685 | 686 | 688 | 924 | 852 |

# 7 Study limitations

The main limitation of this study is the lack of experimental data to confirm the observations from the simulations. There are very few experimental methods to study the dynamics of RNA-protein complexes and many of the require labeling of one or other of the biomolecules which might disturb the system. Furthermore, the state-of-the-art MD simulations are generally run for tens or even hundreds of microseconds. We acknowledge, that our total simulation time of 1 or 2 μs per force field could lack sampling. However, seeing that our data generally follows normal distribution (Figures 2-3), some assumptions can be made, and we are able to provide a short overview of different force fields in the simulation of RNA-protein complexes. As the choice of force field seems to be case-dependent, researchers can follow a similar procedure of relatively short simulations to choose a suitable force field for their RNA-protein complex. The water models differ between the different force fields, as we tried to follow the recommendations of the force field publications. Some of the differences observed could be due to the differences in the water models instead of the force field.

Another commonly used non-polarizable force field CHARMM36 was not included in this study, as results of RNA-protein complex simulations with it in comparison with the Amber force fields are presented in another very recent study (Gallardo et al., 2022). In that study, CHARMM36 simulations showed lower hydrogen bond count than the Amber simulations and less interaction between the RNA and the protein. The studied system had the RNA bound on the protein surface which allowed freer movement for it than the Ago2 binding site which is in-between of the protein domains. Based on the CHARMM36 results and our results with OPLS4, it seems that the Amber force fields result in higher hydrogen bond count and less fluctuating complex.
